# O Ano de 2021 na Pesquisa Básica: a Procura por Modelos Translacionais

**DOI:** 10.36660/abc.20220186

**Published:** 2022-04-07

**Authors:** Mariana Gatto, Gustavo Augusto Ferreira Mota, Marina Politi Okoshi

**Affiliations:** 1 Faculdade de Medicina de Botucatu Universidade Estadual Paulista Botucatu SP Brasil Faculdade de Medicina de Botucatu - Universidade Estadual Paulista, UNESP, Botucatu, SP – Brasil

**Keywords:** Doenças Cardiovasculares, Pesquisa Experimental, Projetos de Pesquisa, Remodelação Ventricular, Insuficiência Cardíaca, Exercício, Pesquisa Médica Translacional

Anualmente, as doenças cardiovasculares (DCV) são responsáveis por aproximadamente 19 milhões de mortes globalmente.^1^ No Brasil, são responsáveis por cerca de um terço das mortes.^2^ Nos últimos anos, houve significativo avanço na medicina cardiovascular. Entretanto, a via final comum das agressões sobre o coração, a insuficiência cardíaca, permanece com elevada incidência, prevalência, e mortalidade.^3^ Portanto, há necessidade de melhor entendimento das DCV e o desenvolvimento de novas abordagens farmacológicas e não farmacológicas para seu tratamento. Em 2021, os Arquivos Brasileiros de Cardiologia publicaram artigos na área de ciências básicas que estiveram, em sua maioria, relacionados a modelos experimentais com elos para futura abordagem translacional para ampliar a compreensão do tratamento das DCV. Neste Editorial, apresentamos uma visão geral de artigos publicados recentemente com ênfase em modelos experimentais para futura abordagem translacional.

Os mecanismos moleculares envolvidos no desenvolvimento da remodelação cardíaca ainda são amplamente investigados.^4^ Os micro-RNAs (miRNA) participam do controle das principais funções celulares, como proliferação, diferenciação, apoptose, resposta ao estresse e regulação transcricional. Em elegante estudo, Xu e Fang^5^ observaram que a expressão do miR-34a e miR-125b é reduzida no coração de pacientes com cardiomiopatia diabética no momento do transplante. Adicionalmente, os autores mostraram, em cultura primária, que aumento da expressão destes miRNAs previne a morte de cardiomiócitos induzida por hiperglicemia.

Apoptose mediada por hipoxemia é importante causa de perda de miócitos e injúria miocárdica. O fator de crescimento endotelial vascular (VEGF) tem sido testado para melhorar a perfusão tecidual. Apesar do interesse na terapia gênica com o VEGF, seus efeitos não estão completamente esclarecidos. Zhang et al.,^6^ mostraram que aumento da expressão do VEGF121 melhora a resposta à hipoxemia de cardiomiócitos de ratos recém nascidos em cultura celular. Condicionamento isquêmico é o processo pelo qual repetidas aplicações de curtos períodos de isquemia alternados com reperfusão induz proteção miocárdica contra insultos isquêmicos de maior duração.^7^ Apesar de intensa investigação, não há fármacos disponíveis para prevenir ou atenuar a lesão induzida por isquemia/reperfusão. Chen et al.,^8^ observaram que a dexmedetomidina, agonista do receptor α2-adrenérgico principalmente utilizada em analgesia e sedação, atenua a lesão induzida por isquemia/reperfusão. O efeito foi caracterizado por melhora da função cardíaca e redução da área infartada. A melhora foi associada a diminuição da apoptose miocitária e inibição da expressão de proteínas da via apoptótica PERK/eIF2α/TCF-4/CHOP. Redução da proteína GRP78, importante marcador de estresse do retículo endoplasmático, foi também observada.

Entre as terapias não farmacológicas, o exercício físico se destaca na prevenção e tratamento das DCV. A pesquisa básica e translacional tem focado nos mecanismos envolvidos nos efeitos benéficos do exercício.^9-11^ Vários estudos mostraram que o exercício melhora a remodelação cardíaca induzida por infarto do miocárdio extenso.^12^ Souza et al.,^10^ observaram que, também em situação de agressão cardíaca menos grave como no infarto do miocárdio pequeno, o exercício aeróbio em esteira, por 12 semanas, melhora a capacidade funcional e preserva a geometria ventricular esquerda. Da mesma maneira, o exercício resultou em efeitos benéficos em ratos com hipertensão renovascular.^11^ Exercício resistido por 12 semanas aumentou a atividade de enzimas antioxidantes e reduziu o dano oxidativo cardíaco e renal, caracterizado por diminuição da concentração de peróxido de hidrogênio e preservação da concentração de grupos sulfidrílicos.^11^

A suplementação com compostos naturais nas doenças cardiovasculares vem ocupando espaço no meio científico, devido à fácil aquisição e baixo custo e toxicidade. A L-carnitina atua no deslocamento de ácidos graxos para sítios de oxidação mitocondrial. A suplementação de L-carnitina reduziu a expressão de genes envolvidos na inflamação, tanto no coração como no tecido adiposo de camundongos diabéticos.^13^ Resultados inovadores foram observados com extrato bruto da planta *Sauromatum guttatum* em ratos Sprague-Dawley com hipertensão arterial induzida por ingestão excessiva de sal. A administração do extrato reduziu a pressão arterial e preservou a função endotelial; em aorta isolada de ratos normotensos, o extrato promoveu o relaxamento vascular.^14^ A ingestão de óleo de copaíba por ratos com hipertensão arterial pulmonar foi acompanhada por efeito antioxidante sistêmico, redução da resistência vascular, e melhora da função do ventrículo direito.^15^ Embora os efeitos anti-inflamatórios e antioxidantes do suco de laranja sejam conhecidos há longo tempo, não havia estudo sobre seu efeito na remodelação cardíaca induzida por infarto do miocárdio. Oliveira et al.,^16^ observaram que a suplementação com suco de laranja aumenta a expressão da heme-oxygenase-1, enzima crucial na homeostasia celular com efeitos anti-inflamatórios, antioxidante e anti-apoptótico.

A deficiência de vitamina D está associada a maior risco de desenvolver DCV, doenças imunes crônicas, e câncer. Entretanto, sua suplementação para prevenção e controle de doenças crônicas e DCV não tem mostrado benefícios.^17,18^ Santos et al.,^19^ observaram que a administração de doses não hipercalcêmicas de vitamina D para ratos normais foi associada a alterações metabólicas e aumento do estresse oxidativo cardíaco.

A doxorubicina é um potente agente antitumoral da família das antraciclinas, amplamente utilizada na terapia antineoplásica. No entanto, seu uso pode resultar em efeitos cardiotóxicos como modulação de proteínas heme, danos ao DNA, e cardiomiopatia.^20,21^ Consequentemente, há grande interesse na descoberta de agentes que possam reduzir a toxicidade da doxorubicina. Brito et al.,^22^ avaliaram o efeito do resveratrol, um componente polifenólico, em cardiomiócitos de ratos recém nascidos tratados com doxorubicina. Os pesquisadores observaram que cardiomiócitos de neonatos cujas mães haviam sido suplementadas na gestação com resveratrol tiveram aumento da viabilidade, da atividade antioxidante e da proteção contra danos gênicos após a adição de doxorubicina.

A pesquisa básica experimental permite grande avanço na compreensão de mecanismos moleculares e celulares envolvidos na performance cardíaca em situações fisiológicas e patológicas. Entretanto, ainda há um longo caminho até que tratamentos farmacológicos e não farmacológicos promissores possam chegar a estudos clínicos e, finalmente, incorporar o arsenal terapêutico para o tratamento das doenças cardiovasculares.
