# Supplementary material for: O Ano de 2021 na Pesquisa Básica: a Procura por Modelos Translacionais
Source: Arq Bras Cardiol. 2022 Apr 7;118(4):667–8. [Article in Portuguese] doi: 10.36660/abc.20220186 (PMC9007007; doi:10.36660/abc.20220186)
Supplement: Translation

# The Year in Basic Research 2021: the Search for Translational Models

**Authors:** Mariana Gatto, Gustavo Augusto Ferreira Mota, Marina Politi Okoshi

**Affiliations:** 1 Brazil Faculdade de Medicina de Botucatu - Universidade Estadual Paulista, UNESP, Botucatu, SP – Brazil

**Keywords:** Cardiovascular Diseases, Experimental Research, Research Design, Ventricular Remodeling, Heart Failure, Exercise, Translacional Medical Research

Cardiovascular diseases (CVD) are responsible for approximately 19 million deaths annually in the world.^1^In Brazil, they are involved in one third of deaths.^2^ A significant improvement in cardiovascular medicine has been observed in the last decades. However, cardiac failure, which is the final common pathway following heart injury, remains with a high incidence, prevalence, and mortality.^3^ A better understanding of CVD may allow the development of new pharmacological and non-pharmacological approaches to their treatment. In 2021, the Arquivos Brasileiros de Cardiologia published articles in the area of basic sciences that were mostly related to experimental models. These studies can provide the basis for a translational approach to expand the understanding of CVD treatment. In this Editorial, we present an overview of recently published articles with emphasis on experimental models for a future translational approach.

The molecular mechanisms involved in the cardiac remodeling development are still widely investigated.^4^ Micro-RNAs (miRNA) participate in the control of major cellular functions, such as proliferation, differentiation, apoptosis, stress response, and transcriptional regulation. In an elegant study, Xu e Fang^5^ observed that miR-34a and miR-125b are downregulated in the heart from patients with diabetic cardiomyopathy at the time of transplant. Additionally, *in vitro* data from rat cardiomyocytes showed that miR-125b and miR-34a overexpression prevents hyperglycemia-induced cardiomyocyte death.

Hypoxemia-mediated apoptosis in cardiomyocytes is a major cause of myocardial injury. Treatment with the vascular endothelial growth factor (VEGF) has been tested to improve tissue perfusion. Despite the interest in VEGF-based gene therapy, its effects are not completely understood. By using transfection of VEGF121 into primary rat cardiomyocytes culture subjected to hypoxia, Zhang et al.^6^ showed that VEGF121 positively affects cardiomyocyte proliferation. Ischemic conditioning is a process whereby repeated application of short periods of ischemia alternating with reperfusion protects the myocardium from longer ischemic insults.^7^ Despite extensive investigation, no drugs are available to prevent or attenuate ischemia/reperfusion injury. The α2-adrenergic receptor agonist dexmedetomidine, mainly used in analgesia and sedation, attenuated ischemia/reperfusion injury in rats by improving cardiac function and reducing infarcted area.^8^ The improvement was associated with decreased myocyte apoptosis and inhibited expression of proteins of the apoptotic pathway PERK/eIF2α/TCF-4/CHOP. A reduction in GRP78 protein, a marker of endoplasmic reticulum stress, was also observed.

Physical exercise is the most important non-pharmacological tool to prevent and treat CVD. Basic and translational research has focused on the mechanisms involved in the benefits of exercise.^9-11^ Several studies have shown that exercise improves cardiac remodeling induced by extensive myocardial infarction.^12^ Souza et al.^10^observed that, also in a condition of slight cardiac aggression, such as in small size infarction, aerobic exercise on a treadmill for 12 weeks improves functional capacity and preserves left ventricular geometry. Likewise, physical exercise had positive effects in rats with renovascular hypertension.^11^ Resistance exercise for 12 weeks increased the activity of antioxidant enzymes and reduced cardiac and renal oxidative damage, characterized by decreased hydrogen peroxide concentration and preserved sulfhydryl groups levels.^11^

The role of natural compounds on the pathophysiology of CVD has attracted the interest of scientists due to its large availability, and low cost and toxicity. L-carnitine is essential to displace fatty acids to mitochondrial oxidation sites. L-carnitine supplementation was shown to reduce the expression of genes involved in inflammation, both in the heart and adipose tissue in diabetic mice.^13^ Innovative results were observed with a crude extract of the plant *Sauromatum guttatum* in Sprague-Dawley rats with arterial hypertension induced by excessive salt intake. The administration of the crude extract reduced blood pressure and preserved endothelial function; in aorta isolated from normotensive rats, the extract promoted vascular relaxation.^14^ Copaiba oil intake by rats with pulmonary arterial hypertension was accompanied by a systemic antioxidant effect, reduced vascular resistance, and improved right ventricular function.^15^ Although the anti-inflammatory and antioxidant effects of orange juice have been known for a long time, there was no study on its effect on infarction-induced cardiac remodeling. Oliveira et al.^16^ observed that dietary supplementation with orange juice increases the expression of heme-oxygenase-1, a crucial enzyme in cellular homeostasis with anti-inflammatory, antioxidant and anti-apoptotic effects.

Vitamin D deficiency is associated with increased risk of developing CVD, chronic immune disease, and cancer. However, its supplementation for prevention and control of chronic diseases and CVD has not shown benefits.^17,18^ Santos et al.^19^ observed that administration of non-hypercalcemic doses of vitamin D to normal rats was followed by metabolic changes and increased cardiac oxidative stress.

Doxorubicin is a potent antitumor agent of the anthracycline family, widely used in anticancer therapy. However, its use can result in cardiotoxic effects such as modulation of heme proteins and DNA damage, and cardiomyopathy.^20,21^ Currently, there is great interest in agents that can reduce the doxorubicin toxicity. Brito et al.^22^ evaluated the effects of resveratrol, a polyphenolic component, on cardiomyocytes from newborn rats treated with doxorubicin. Myocytes from neonates whose mothers had been supplemented during pregnancy with resveratrol had increased viability, antioxidant activity, and protection against gene damage after the addition of doxorubicin.

Basic experimental research allows great advances in the understanding of molecular and cellular mechanisms involved in cardiac performance in physiological and pathological conditions. However, there is still a long way before promising pharmacological and non-pharmacological treatments can be tested in clinical studies and finally incorporated into the therapeutic arsenal currently available for the treatment of cardiovascular diseases.
